# Germline copy number variants are not associated with globally acquired copy number changes in familial breast tumours

**DOI:** 10.1007/s10549-012-2024-6

**Published:** 2012-03-21

**Authors:** Logan C. Walker, Lutz Krause, kConFab Investigators, Amanda B. Spurdle, Nic Waddell

**Affiliations:** 1 Department of Pathology, University of Otago, Christchurch, 8011 New Zealand; 2 Queensland Institute of Medical Research, PO Royal Brisbane Hospital, Brisbane, QLD 4029 Australia; 3 Peter MacCallum Cancer Centre, St Andrews Place, East Melbourne, VIC 3002 Australia; 4 Queensland Centre for Medical Genomics, Institute for Molecular Biosciences, University of Queensland, Brisbane, QLD Australia

**Keywords:** Copy number variant, Familial breast cancer, Tumour genome, SNP array

## Abstract

A characteristic of sporadic and familial breast tumours is genomic instability, resulting from either inherited mutations in genes that control genome integrity or mutations that are acquired in somatic cells during development. It is well established that abnormal chromosome number and structural changes to chromosomes play an important role in the cause and progression of breast cancer. Familial *BRCA1* breast tumours are characterised by basal-like phenotype and high-histological grade which are typically associated with increased genomic instability. Consistent with previous studies, the genomes with the greatest number of base pairs covered by copy number change were typically found in basal-like and/or high-histological grade breast tumours within our cohort. Moreover, we show that luminal A tumours that are high grade had significantly less copy number variant (CNV) coverage than the more clinically aggressive high-grade luminal B tumours, suggesting that chromosomal instability rather than cellular differentiation contributes to the aggressive nature of luminal B tumours. It has previously been proposed that germline CNVs may contribute to somatically acquired chromosome changes in the tumour, but this is the first study to address this idea in breast cancer. By comparing germline CNVs and tumour-specific CNVs in matched breast tumour and normal tissue using data from the Illumina Human CNV370 duo beadarray, we provide evidence that germline CNVs do not tend to act as a foundation on which larger chromosome copy number aberrations develop in tumour cells. Further studies are required with increased sequence resolution that will detect smaller CNVs and define CNV breakpoints to comprehensively assess the relationship between inherited genomic variation and genome evolution in breast cancer.

**Electronic supplementary material:**

The online version of this article (doi:10.1007/s10549-012-2024-6) contains supplementary material, which is available to authorized users.

## Introduction

Familial and sporadic forms of breast cancer are now recognised to be a complex and heterogeneous disease at both the clinical and molecular levels [1–4]. The genome of a breast tumour typically represents a culmination of somatically acquired, poorly understood, genomic aberrations that functionally alter genes contributing to tumourigenesis [5]. The extent of genomic abberation in breast cancer has been shown to differ significantly in histological subtype, such as low- and high-grade tumours [6, 7]. Since patients with high-grade tumours generally have worse prognosis than those who have low-grade tumours [8], these findings indicate that there is an association between the mutational burden of tumours and tumour pathogenesis. Similarly, basal-like breast tumours are characterised by high levels of genomic aberrations in comparison to other tumour subtypes, such as the luminal A or luminal B subtypes [1], and are associated with poor prognosis [9]. A number of phenotypical and molecular features are shared by basal-like breast cancer and tumours arising in *BRCA1* germline mutation carriers, including high grade and a high number of chromosome copy number changes [10]. This suggests a common tumourigenic pathway of the *BRCA1* and basal-like subtypes; however, the biological mechanisms associated with increased frequency of chromosomal changes in these tumour types are currently poorly understood.

Studies of choroid plexus tumours in Li–Fraumeni Syndrome (LFS)-affected families [11] and of colon cancer-affected individuals [12] have suggested that constitutional copy number variants (CNVs) may act as a foundation on which chromosome copy number aberrations develop in tumour cells. These findings suggest a direct relationship between constitutional genomic variation and tumour genome evolution. However, the number of cancer cases, where matched normal tissue was also assessed, was relatively small for each of the choroid plexus tumour and colon cancer studies (*n* = 4 and 5, respectively) [11, 12]. Furthermore, it is currently unknown whether germline CNVs play a role in genomic instability associated with breast tumour grade and breast tumour subtype. To address this issue, we utilised single nucleotide polymorphism array data from a previously published study [1] to compare germline CNV and breast tumour-specific CNVs using 28 matched normal and tumour tissue pairs. Furthermore, we reassessed the association between pathological features, such as breast tumour subtype and histological grade, and the extent of CNV coverage in germline and tumour genomes.

## Methods and materials

### Patient material

Twenty-eight breast cancer affected women participating in this study were from multi-case families who had been recruited into the Kathleen Cuningham Foundation for Research into Breast Cancer (kConFab) [13]. Of these, 9 carried a mutation in *BRCA1*, 7 carried a mutation in *BRCA2* and 12 were mutation negative (BRCAx) after full sequencing and multiplex ligation-dependant probe amplification (MLPA) analysis of *BRCA1/2*. The histological features of all tumours were reviewed by a pathologist who also scored the percentage of neoplasia in the specimen prior to DNA isolation, as previously described [1]. The molecular subtype (luminal A, luminal B, HER2, basal and normal breast-like) for each tumour had been earlier determined by Waddell et al. [1].

### Genome-wide SNP genotyping

SNP genotyping data were generated from 28 matched tumour and normal tissue using Illumina arrays containing 370,000 SNPs. Tissue samples of the primary tumour and matching normal tissue were obtained by macrodissectioning frozen sections of 10 μm with a needle, ensuring that the neoplastic content was >75%. DNA was extracted by the salting-out method followed by a phenol chloroform extraction. The Infinium II assay protocol (Illumina Inc., San Diego, CA) was used to perform whole genome amplification. DNA of 750 ng was fragmented and hybridised to Illumina Human CNV370 duo beadarrays [1].

### CNV calling and data analysis

Germline and tumour-specific CNVs were determined using the SNP array data for all 28 matching tissue pairs. Data were imported and visualised in GenomeStudio Software v2010.3, and the B-allele frequencies and logR ratios were exported for each sample. The software package R was used to perform SOMATICS [14] to identify regions containing CNVs, as previously described [1]. SOMATICS was used as it can analyse SNP data from tissues which are heterogeneous due to the presence of stromal contamination or multiple tumour clones. For technical validation of predicted CNVs, comparative qPCR was performed as described previously [1]. Galaxy genome analysis tools [15] were utilised for mapping CNVs to the hg18 build of the human genome and to perform intersection and subtraction, to compute base coverage and to obtain flanking sequences.

In this study, we defined tumour-specific CNV regions as those which show copy number change in the tumour genome but do not overlap germline CNV regions identified in the genome of the matched normal tissue. To account for the possibility that contiguous CNVs called by SOMATICS may represent a single larger CNV, especially in the tumour genome, we have measured the base pair coverage of these variants as opposed to their frequency. Our hypothesis that germline CNVs are ‘hotspots’ for tumour-specific CNVs, and hence that the fraction of tumour-specific CNVs located in proximity to germline CNVs is higher than expected was tested in silico. First, we estimated the expected fraction of genomic DNA containing tumour-specific CNVs in proximity to germline CNVs. For each sample, mock CNVs of the same size as the observed set of tumour-specific CNVs were randomly distributed over the human genome. Using these data, we computed the fraction of randomly placed CNVs in proximity to a germline CNV (overlapping or less than 1,000 bp downstream or upstream). This simulation was repeated 2,000 times for each sample. The average fraction of randomly placed CNVs in proximity to a germline CNV was used as the expected fraction. Second, we tested the hypothesis ‘the fraction of tumour-specific CNVs located in proximity to a germline CNV is higher than expected’ with the paired-Wilcoxon rank test (*P* < 0.05). Genomic coordinates corresponding to germline CNVs and tumour-specific CNVs used in this analysis are listed in Table S1 (Supplementary material). A schematic representation of the experimental design is illustrated in Fig. S1 (Supplementary material).

## Results

### DNA copy number profiles of matched breast tumour and normal tissue pairs

The germline DNA copy number profiles of our study cohort show that on average, 2.6% (range 0.6–7.0%) of a haploid genome was affected by copy number change (Table S2, Supplementary material). By comparison, analysis of DNA from matched breast tumour tissue revealed copy number changes that covered 37.6% (range 10.5–70.1%) of the haploid genome (Table S2, Supplementary material) and tumour-specific copy number (germline CNV regions excluded) changes covering 36.4% (range 10.0–69.3%) of the haploid genome. These results therefore show that germline CNVs covering an average of 1.4% of the genome are not detected in the tumour genome. There are several potential explanations for this difference, including the possibility that (1) the genomic regions containing germline CNVs may change copy number status after further rearrangement of the tumour; (2) the complexity of the structural rearrangements in the tumour genome results in a failure to call some tumour CNVs by SOMATICS and (3) a proportion of CNVs in the germline DNA are miscalled.

No significant difference was observed in the number of base pairs affected by germline CNVs when comparing cases by mutation status, histological grade of the tumour and basal/non-basal subtype (Table 1). However, a comparison of germline CNV base pair coverage between cases with luminal A and luminal B tumours did show a twofold increase in coverage for luminal A cases that was moderately significant (*P* = 0.04; Table 1).

**Table 1:** Genomic coverage of germline and tumour-specific CNV regions

	*n*	Germline CNV regions		Tumour-specific CNV regions	
		Average base pair coverage	*P* ^a^	Average base pair coverage	*P* ^a^
Mutation status					
*BRCA1*	9	79,801,545	0.70_(*BRCA1* vs *BRCA2*)_	1,359,740,736	0.03_(*BRCA1* vs *BRCA2*)_
*BRCA2*	7	70,879,436	0.52_(*BRCA2* vs BRCAx)_	890,264,516	0.39_(*BRCA2* vs BRCAx)_
BRCAx	12	84,758,924	0.84_(*BRCA1* vs BRCAx)_	1,092,785,789	0.23_(*BRCA1* vs BRCAx)_
Molecular subtype					
Basal	13	68,410,342	0.27_(Basal vs all other subtypes)_	1,560,362,706	10^−7^_(Basal vs all other subtypes)_
Her2	2	73,290,002		1,141,773,431	
Luminal A	8	114,241,923		550,866,350	
Luminal B	4	57,603,536	0.04_(LumA vs LumB)_	1,074,264,196	10^−4^_(LumA vs LumB)_
Normal	1	51,213,066		310,698,090	
Histological grade					
Grade II	6	111,118,574		658,829,402	
Grade III	21	73,035,723	0.20_(Grade II vs Grade III)_	1,245,855,304	0.007_(Grade II vs Grade III)_

^a^Two-tailed Student’s *t* test

**Fig. 1:**
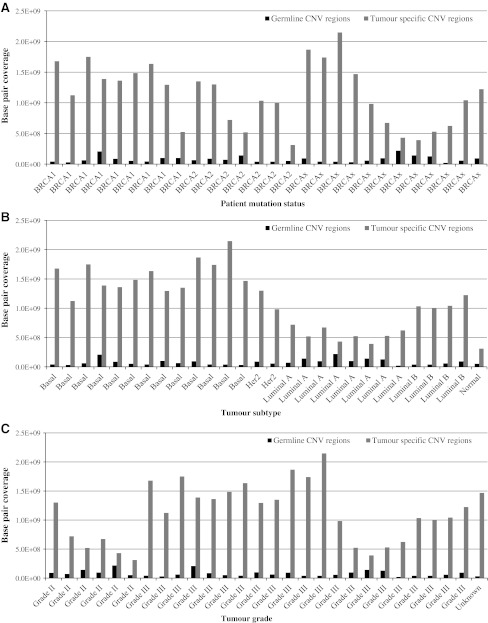
Base pair coverage of germline and tumour-specific CNV regions as stratified by **a** patient mutation status, **b** breast tumour subtype and **c** breast tumour grade

In contrast to germline CNVs, and as previously demonstrated [1], the average number of base pairs affected by copy number change within the tumours differed significantly between molecular subtypes and to a lesser degree, by mutation status (Fig. 1a, b; Table 1). This difference was most striking (twofold, *P* = 10^−7^) when comparing genomic profiles from basal and non-basal breast tumours (Fig. 1b; Table 1). The average number of base pairs affected by copy number change also differed significantly (twofold, *P* = 0.007) when classified by histological grade (Fig. 1c; Table 1). This finding is concordant with previous studies that have reported the frequency of genomic aberrations in grade III breast tumours to be greater than those found in grade I or II tumours [7, 16–18]. Sample numbers limited the number of statistical comparisons that could be carried out between the various molecular subtypes. However, we were able to compare the genomic profiles of luminal A and luminal B tumours, and found that the CNV coverage in luminal B tumours was twofold greater than that in luminal A tumours (*P* < 10^−4^; Table 1). This difference is not explained by the observation that four of the eight luminal A tumours were also grade II, as a comparison of luminal A and luminal B grade III tumours also showed a significant difference in base pair coverage (*P* = 0.0002).

### Correlating the location of germline CNVs with tumour-specific CNVs in paired tumour tissue

**Fig. 2:**
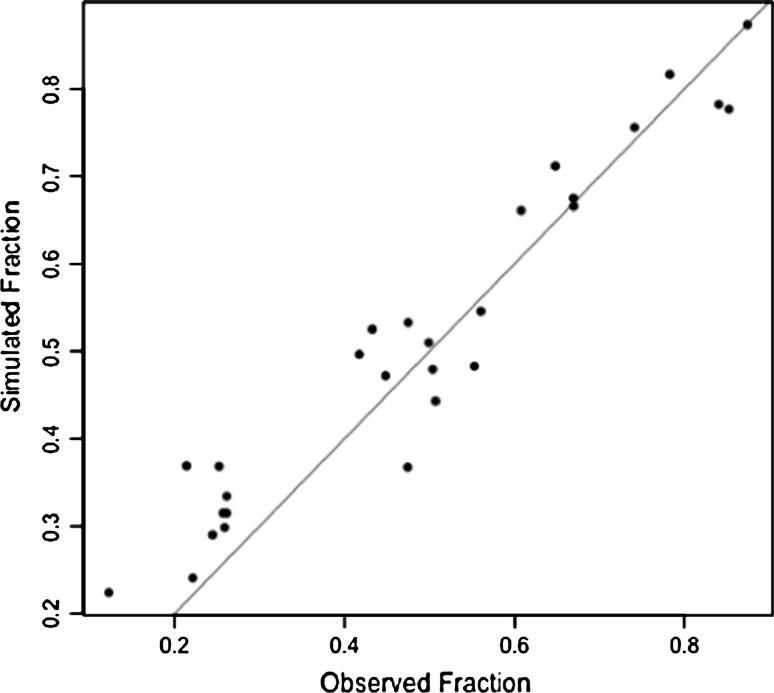
Proximity of tumour-specific CNVs to germline CNVs from actual and simulated data. Each dot represents for one patient the fraction of observed and expected (randomly placed) tumour CNVs in proximity to a germline CNV

To assess whether germline CNVs act as ‘hotspots’ for tumour-specific CNVs, we tested the hypothesis that tumour-specific CNVs are preferentially located in proximity to germline CNVs. On average, 18.8% (range 1.8–38.7%) of haploid genomes from the breast tumours evaluated in this study consisted of tumour-specific CNVs that either overlapped or were located within 1 kb of a germline CNV. This equates to approximately half of the total number of base pairs that are affected by tumour-specific CNVs (36.4%, range 10.0–69.3%). To determine whether these germline CNVs are hotspots for tumour-specific CNVs, we simulated events using observed CNV sizes and calculated the fraction of randomly placed tumour-specific CNVs in proximity to a germline CNV. We found that the overall difference between actual and simulated events was not significant (*P* = 0.97; Fig. 2; Table 2). Only 10 of 28 cases showed greater proximity between the actual germline CNVs and tumour-specific CNVs and represented all tumour subtypes (Table 2). By comparison, the proximity between randomised intervals of germline CNVs and tumour-specific CNVs in 18 of the 28 cases was slightly greater than that between actual intervals of germline CNVs and tumour-specific CNVs (Table 2). This was also the case for 9 of the 13 basal-like tumours suggesting that germline CNVs in these tumours are not located in genomic regions that give rise to somatically acquired changes. Together these findings suggest that the location of tumour-specific CNVs is not biased by germline CNVs.

**Table 2:** Proximity of tumour-specific CNVs to germline CNVs from actual and simulated data (2000 replications per sample)

Sample ID	Mutation status	Original bp fraction in proximity (*x*)	Simulated bp fraction in proximity			Difference (*x* − *y*)
			Average fraction (*y*)	Minimum fraction	Maximum fraction	
B7	BRCA1	0.78	0.82	0.69	0.90	−0.03
B8	BRCA1	0.65	0.71	0.44	0.84	−0.07
B9	BRCA1	0.12	0.22	0.10	0.38	−0.10
B11	BRCA1	0.47	0.37	0.20	0.53	0.11
B15	BRCA1	0.43	0.52	0.35	0.68	−0.09
B16	BRCA1	0.67	0.67	0.43	0.82	0.00
B19	BRCA1	0.51	0.44	0.23	0.64	0.06
B21	BRCA1	0.25	0.36	0.16	0.54	−0.11
B22	BRCA1	0.50	0.48	0.30	0.64	0.03
B2	BRCA2	0.47	0.54	0.37	0.74	−0.06
B3	BRCA2	0.26	0.34	0.08	0.54	−0.08
B5	BRCA2	0.50	0.50	0.27	0.67	−0.01
B12	BRCA2	0.22	0.25	0.04	0.44	−0.02
B13	BRCA2	0.42	0.50	0.27	0.69	−0.08
B17	BRCA2	0.21	0.38	0.00	0.71	−0.16
B27	BRCA2	0.84	0.78	0.62	0.86	0.06
B1	BRCAx	0.87	0.87	0.70	0.92	0.00
B4	BRCAx	0.24	0.29	0.13	0.45	−0.04
B6	BRCAx	0.74	0.75	0.54	0.84	−0.01
B10	BRCAx	0.61	0.66	0.52	0.77	−0.05
B14	BRCAx	0.26	0.32	0.10	0.54	−0.06
B18	BRCAx	0.56	0.55	0.30	0.74	0.01
B20	BRCAx	0.67	0.67	0.50	0.79	0.00
B23	BRCAx	0.55	0.47	0.03	0.70	0.08
B24	BRCAx	0.85	0.78	0.62	0.87	0.08
B25	BRCAx	0.45	0.47	0.28	0.64	−0.02
B26	BRCAx	0.26	0.29	0.08	0.49	−0.03
B28	BRCAx	0.26	0.32	0.00	0.54	−0.06

*bp* Base pair

## Discussion

Similar to sporadic breast tumours, familial breast tumours can be classified into at least five molecular subtypes that are clinically distinct [1]. Of these subtypes, the basal-like tumour subtype, which is common in *BRCA1* mutation carriers, is characterised by a high number of chromosomal copy number changes. Genomic instability is also a molecular feature of tumours with high-histological grade that, analogous to basal-like tumours, are typically aggressive lesions. The ability to predict which breast cancer patients will develop tumours with extensive genomic instability will undoubtedly be a valuable tool in clinical diagnostics. To our knowledge, this is the first study to test the hypothesis that variation in genotype as a result of inherited copy number changes contributes to genomic instability within breast tumour cells. In contrast to the previous small studies [11, 12] that evaluated choroid plexus and colon carcinomas, our results using 28 matched breast tumour and normal tissue suggest that, at a whole genome level, there is no evidence of an association between the genomic location of germline CNVs and breast tumour-specific CNVs using matched normal and tumour pairs.

Consistent with a previous study of familial breast tumours, we found that CNV base pair coverage in luminal B tumours was significantly greater than that in luminal A tumours [19]. However, we show that this difference was independent of breast tumour grade. Compared to luminal A tumours, luminal B tumours are known to have higher cellular proliferation and confer poorer prognosis [20]. Our results suggest that the chromosomal instability phenotype, but not the differentiated state of the tumour cells, contributes to the aggressive nature of luminal B tumours.

A previous study found a significant association between known CNV loci and de novo chromosome breaks in colon cancer [12], suggesting that germline CNVs and tumour-specific CNVs are likely to be located at chromosome regions that are most prone to breakage. However, it remains to be determined whether sequences at these loci predispose to further genomic instability after copy number change has occurred in the germline DNA. Our study went some way to investigate this issue; however, there are notable limitations with the current dataset. First, the exact boundaries of predicted CNVs cannot be precisely determined using microarray-based platforms, and it is therefore impossible to characterise the breakpoint sequences that are involved in the copy number change without further sequence analysis. Second, it is unclear as to the precise location of duplicated or amplified CNVs in that some CNV units may not be located in tandem but may map to different chromosomes entirely; confounding studies assessing their contribution to somatically acquired genomic events. Whole genome high-resolution technologies, such as next-generation sequencing, will be required to accurately map the location of each CNV amplicon to better ascertain which sequences contribute to tumour genome instability. Third, SOMATICS was not able to discriminate heterozygous loss and gain for some predicted CNVs. Thus, we were only able to identify copy number variable regions that had undergone copy number change but were unable to classify many of these regions by exact copy number status. Forth, germline DNA used by Waddell et al. [1] was obtained from breast tissue that appears histologically normal but may potentially harbour somatically acquired copy number changes. Although DNA from peripheral blood cells or buccal cells would have prevented such possibility, recent evidence suggests that the detection of clonal changes in normal tissue found adjacent breast tumours would be unlikely [21, 22].

The notion that inherited CNVs may influence incidence of the various genomic copy number changes that occur during breast cancer progression has not only prognostic significance, but also important consequences for early decisions relating to clinical management. Although our findings suggest no association globally across the genome, it is still possible that some germline CNVs may indeed mark regions that are prone to further rearrangement in the breast tumour. Further work is therefore required using the latest genomic sequencing technologies to precisely map CNV breakpoints sequences across the genome to determine the relationship between inherited genomic variation and genome evolution in breast cancer. Moreover, studies with larger cohort size are warranted to assess our finding in familial breast tumours, that the genome of high-grade luminal A tumours had significantly less CNV coverage than the more clinically aggressive high-grade luminal B tumours.

## Electronic supplementary material

Below is the link to the electronic supplementary material.

Supplementary material 1 (DOC 509 kb)

Supplementary material 2 (XLSX 511 kb)

Supplementary material 3 (XLSX 13 kb)
