# Supplementary material for: Germline copy number variants are not associated with globally acquired copy number changes in familial breast tumours
Source: Breast Cancer Res Treat. 2012 Mar 21;134(3):1005–11. doi: 10.1007/s10549-012-2024-6 (PMC3409366; doi:10.1007/s10549-012-2024-6)
Supplement: Supplementary file 1 — Supplementary material 1 (DOC 509 kb) [file 10549_2012_2024_MOESM1_ESM.doc]

**Figure S1.** Schematic representation of the experimental design linked to a screenshot of UCSC Genome Browser displaying an example of germline CNVs (gCNVs; A), tumour CNVs (tCNVs; B), tumour specific CNVs (tsCNVs; C), and tsCNVs that are located in proximity to gCNVs (tsCNVs/gCNVs; D).


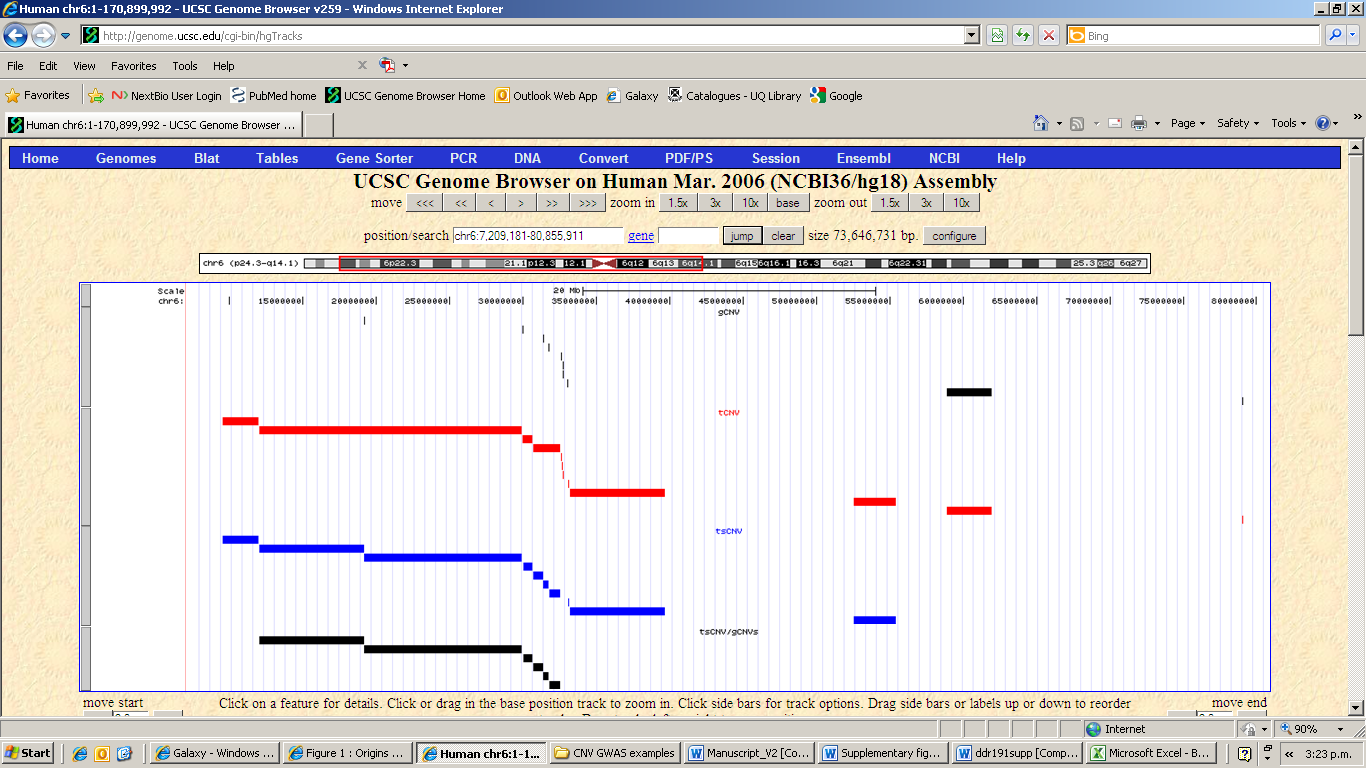


**A**

**B**

**C**

**D**

**CNV discovery (Waddell et al, 2010):** CNV identification in germline DNA and tumour DNA from 28 matched breast tumour and normal tissue pairs using SOMATICS.

gCNVs mapped across the genome (**A** – Genome Browser screenshot)

tCNVs mapped across the genome (**B** - Genome Browser screenshot)

**Identification of tumour specific CNVs (tsCNVs)** - genomic regions that have undergone copy number change in the tumour but not the genome (**C** - Genome Browser screenshot)

Identification of gCNVs that overlap or are located within 1kb of a tsCNV (**D** - Genome Browser screenshot)

**)**.

**Estimating the expected fraction of genomic DNA containing tsCNVs in proximity to gCNVs.**

1) Mock CNVs of the same size as the observed set of tumour specific CNVs were randomly distributed over the human genome for each sample.

2) The fraction of randomly placed CNVs in proximity to a gCNV was computed. This simulation was repeated 2000 times for each sample.

3) Comparison of the observed and expected (simulated) fraction of randomly placed CNVs in proximity to a gCNV.
